# Supplementary material for: Derailed protein turnover in the aging mammalian brain
Source: Mol Syst Biol. 2024 Jan 5;20(2):120–39. doi: 10.1038/s44320-023-00009-2 (PMC10897147; doi:10.1038/s44320-023-00009-2)
Supplement: Supplementary file 5 — Source Data Fig. 4 [file 44320_2023_9_MOESM5_ESM.zip › MSB202311808_SourceDataforFig4E.pdf]

Source Data Fig. 4E

DUB Activity					
	BR1	BR2	BR3	BR4	Blank
12 M	148059	151071	201321.5	139904	106151.5
15 M	182387.5	246652.5	218484.5	192728.5	
18 M	245939.5	232907.5	302167.5	183358.5	
21 M	182431.5	213372	180953	204953	
24 M	183321.5	157268.5	180233	160798	
