# Supplementary material for: Derailed protein turnover in the aging mammalian brain
Source: Mol Syst Biol. 2024 Jan 5;20(2):120–39. doi: 10.1038/s44320-023-00009-2 (PMC10897147; doi:10.1038/s44320-023-00009-2)
Supplement: Supplementary file 5 — Source Data Fig. 4 [file 44320_2023_9_MOESM5_ESM.zip › MSB202311808_SourceDataforFig4F.pdf]

Source Data Fig.4 F

| 20S Core Activity | | | | | |
|---|---|---|---|---|---|
| | BR1 | BR2 | BR3 | BR4 | Blank |
| 12 M | 479067 | 434603 | 501583.5 | 415943 | 304159.5 |
| 15 M | 540656.5 | 656622.5 | 592930.5 | 528781 | |
| 18 M | 586599.5 | 536103 | | 560989 | |
| 21 M | 628475.5 | 622508.5 | 628445.5 | 606591 | |
| 24 M | 586868.5 | 506513.5 | 584286 | 526066.5 | |
