# Supplementary figures and images for: Derailed protein turnover in the aging mammalian brain
Source: Mol Syst Biol. 2024 Jan 5;20(2):120–39. doi: 10.1038/s44320-023-00009-2 (PMC10897147; doi:10.1038/s44320-023-00009-2)

Source Data Fig. 4B

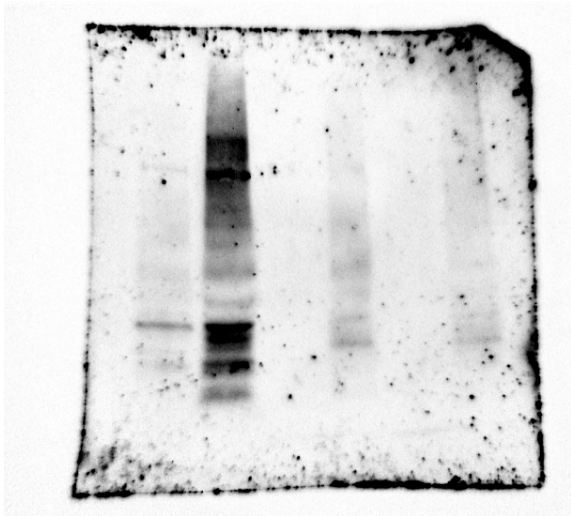

Supplement: Supplementary file 5 — Source Data Fig. 4 [file 44320_2023_9_MOESM5_ESM.zip › MSB202311808_SourceDataforFig4B.pdf]
