# Supplementary material for: Derailed protein turnover in the aging mammalian brain
Source: Mol Syst Biol. 2024 Jan 5;20(2):120–39. doi: 10.1038/s44320-023-00009-2 (PMC10897147; doi:10.1038/s44320-023-00009-2)
Supplement: Supplementary file 6 — Source Data Fig. 5 [file 44320_2023_9_MOESM6_ESM.zip › MSB202311808_SourceDataforFig5C.pdf]

Source Data Fig. 5C

20S Core Activity												
	VEH BR1	VEH BR2	VEH BR3	VEH BR4	VEH BR5	MAR BR1	MAR BR2	MAR BR3	MAR BR4	MAR BR5		Blank
Tech1	702720	703079	715468	831568	977320	592038	603014	718737	520291	654120		216917
Tech2	735080	706779	699266	795906	847926	568927	580272	724723	533824	612216		209895
