# Supplementary material for: Derailed protein turnover in the aging mammalian brain
Source: Mol Syst Biol. 2024 Jan 5;20(2):120–39. doi: 10.1038/s44320-023-00009-2 (PMC10897147; doi:10.1038/s44320-023-00009-2)
Supplement: Supplementary file 6 — Source Data Fig. 5 [file 44320_2023_9_MOESM6_ESM.zip › MSB202311808_SourceDataforFig5D.pdf]

Source Data Fig. 5D

DUB Activity												
	VEH BR1	VEH BR2	VEH BR3	VEH BR4	VEH BR5	MAR BR1	MAR BR2	MAR BR3	MAR BR4	MAR BR5		Blank
Tech1	893625	813958	790549	815490	824727	775787	823329	826066	775141	814846		627292
Tech2	860090	866113	842291	818820	857067	796677	838050	839081	802646	796484		649974
