# Supplementary material for: Derailed protein turnover in the aging mammalian brain
Source: Mol Syst Biol. 2024 Jan 5;20(2):120–39. doi: 10.1038/s44320-023-00009-2 (PMC10897147; doi:10.1038/s44320-023-00009-2)
Supplement: Supplementary file 8 — Expanded View Figures [file 44320_2023_9_MOESM8_ESM.pdf]

Expanded View Figures

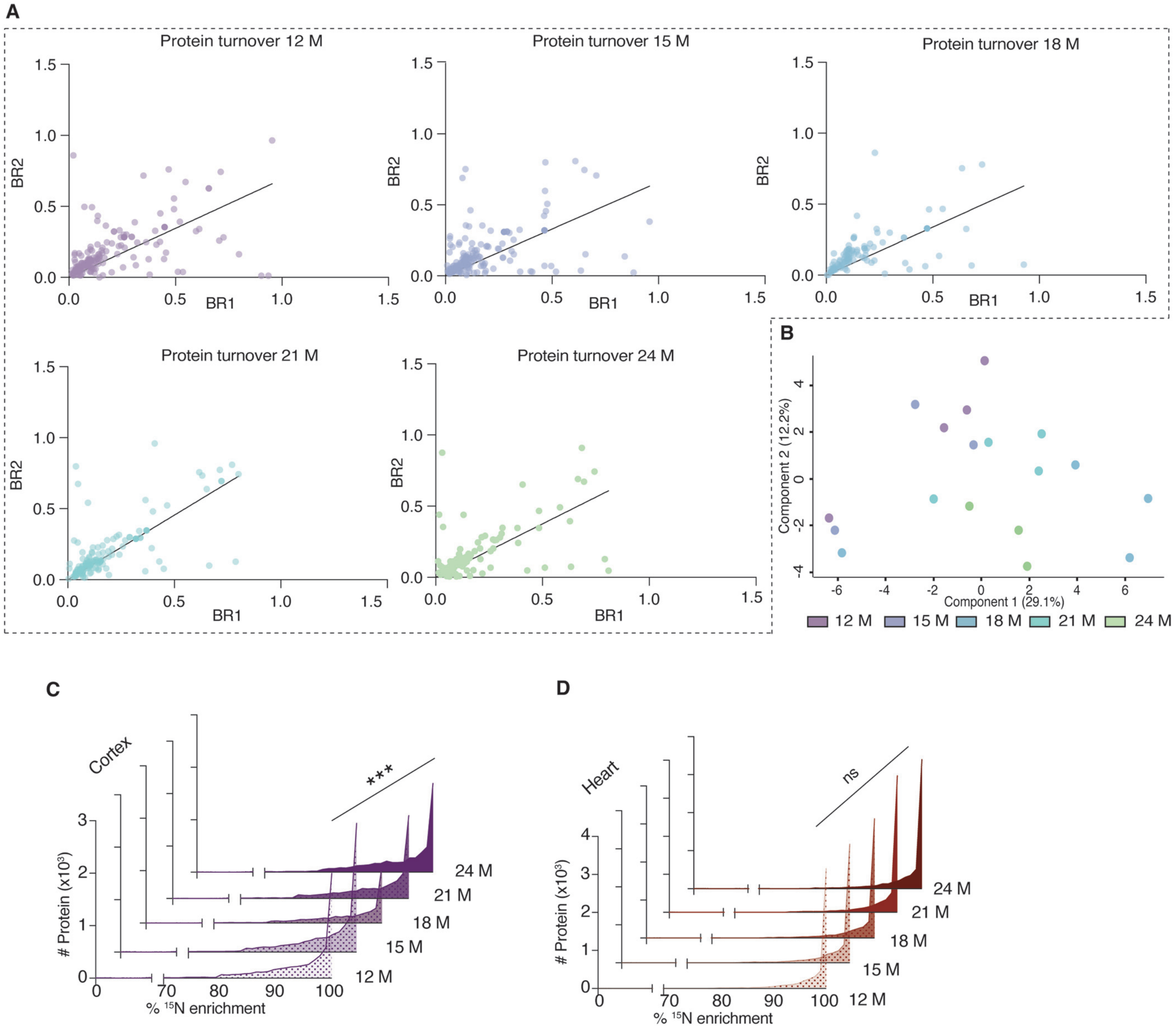

Figure EV1. **^{15}N labeling efficiency and proteome-wide measures of ^{15}N incorporation in cortex and heart.**

A Biological replicates from each age group show reproducible ^{14}N fractional abundance ($^{14}N/(^{14}N + ^{15}N)$) in cortex. **B** PCA analysis of each biological replicate from the 5 age groups. **C, D** ^{15}N incorporation plots for each age group in the cortex and heart showing the proportion of the proteome that was labeled with ^{15}N. All data are mean ± SEM with $n = 3$–4 female mice. **p* value < 0.05; ***p* value < 0.01; ****p* value < 0.001 by Kruskal–Wallis ANOVA with Tukey's multiple comparisons test.

 © The Author(s)

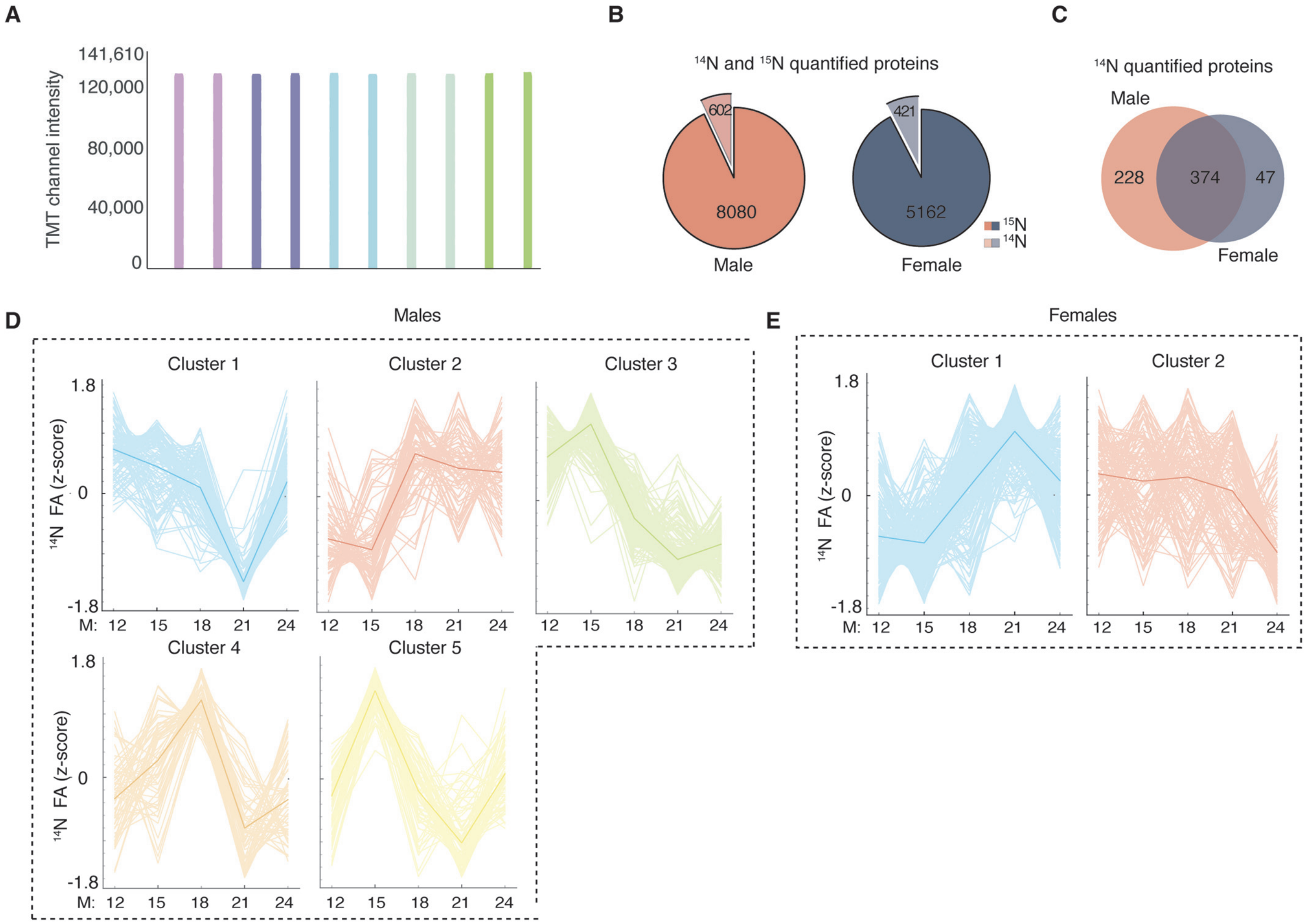

Figure EV2. Quantification of proteins identified from TMT-MS experiments using novel analytical workflow.

A Representative global TMT channel peak intensities for each reporter ion from 10-plex TMT-MS^3 experiment demonstrating equal labeling across all channels. **B** Pie charts depicting quantified ^{14}N and ^{15}N proteins from TMT-MS^3 analysis of male and female datasets. **C** Venn diagram showing the overlap between male and female quantified ^{14}N proteins. **D**, **E** Line plots for each cluster from heatmap showing trends of protein turnover for males and females. All data are mean ± SEM with $n = 4$ mice for both sexes except $n = 3$ for female 15 M and 24 M groups.

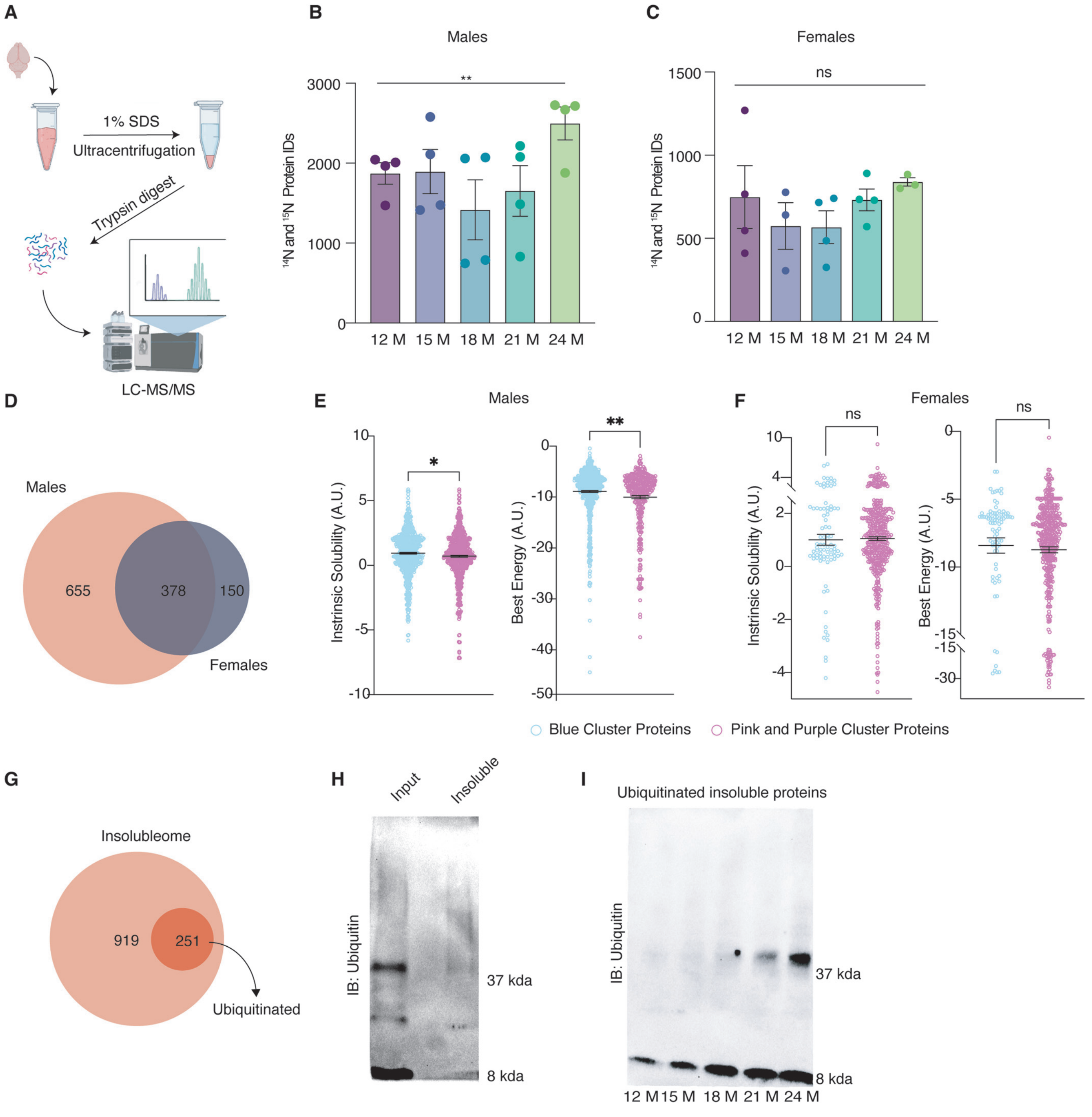

Figure EV3. Confirmation of biochemical isolation and MS-based analysis of insoluble cortical proteome.

A Schematic depicting the biochemical isolation and analytical workflow of the insoluble proteome from cortical extracts. **B, C** Number of protein identifications for each age group in male and female datasets. **D** Venn diagram showing overlap of identified insoluble proteins from male and female datasets. **E, F** Intrinsic solubility and best energy of purple and pink versus blue clusters from male and female datasets. **G** Venn diagram showing number of identified ubiquitinated proteins compared to insolubleome. **H** Western blot showing presence of ubiquitinated proteins in insoluble protein pools. **I** Western blot showing age-dependent increase in ubiquitinated proteins present in the insoluble pool in the indicated age groups in males. All data are mean ± SEM with $n = 4$ mice for both sexes except $n = 3$ for female 15 M and 24 M groups. ^{14}N FA is standardized to median of 12 M group. *p value < 0.05; **p value < 0.01; ***p value < 0.001 by Kruskal–Wallis ANOVA with Tukey's multiple comparisons test or Student's t test.

 © The Author(s)

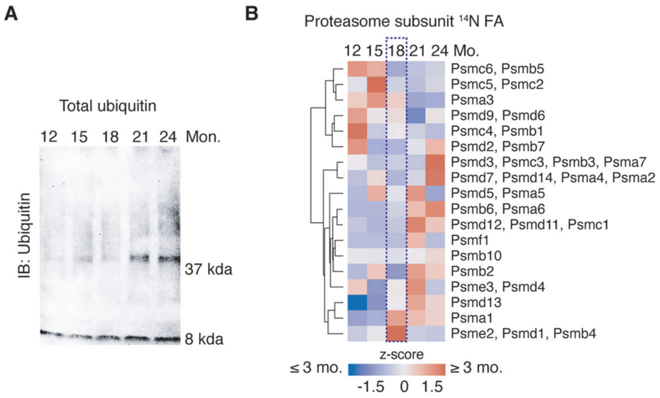

Figure EV4. Ubiquitination and proteasome core turnover profiles.

A Western blot for ubiquitinated protein levels from isolated proteasomes shows increased abundance at 21 M and 24 M. **B** Heatmap representation of quantified proteasome subunit ^{14}N fractional abundance across aging cohorts. All data are mean ± SEM with $n = 4$.

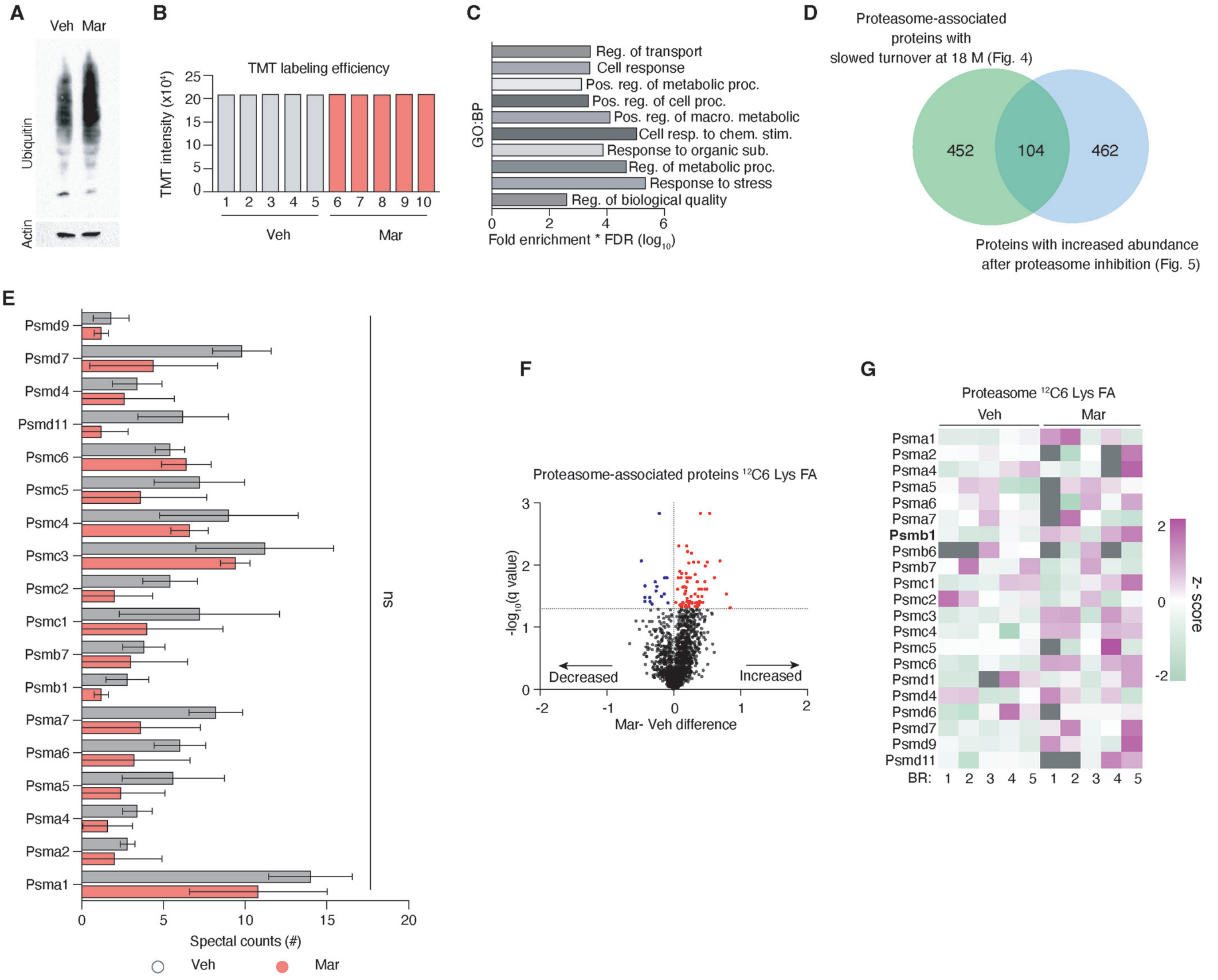

Figure EV5. Detailed effects of partially suppressed proteasome activity with marizomib.

A Western blot confirming accumulation of ubiquitinated proteins after marizomib treatment. **B** Representative global TMT channel peak intensities for each reporter ion from 10-plex TMT-MS^3 experiment demonstrating equal labeling across all channels. **C** Gene ontology enrichment analysis showing the biological processes that are overrepresented in the group of proteins with significantly increased abundance after marizomib. **D** Confirmation that about one-fourth of proteasome subunits and copurifying proteins previously found modulated during aging are similarly modulated by marizomib. $p < 0.001$ by Fisher's exact test. **E** No differences in quantity proteasome subunits proteins identified between vehicle and marizomib cohorts after biochemical proteasome isolation. **F** Significant differences between marizomib and vehicle cohorts is show with red and blue representing increased and decreased ^{12}C6-lysine fractional abundance, respectively. Total $n = 1426$ quantified proteins. **G** Heatmap representation of ^{12}C6-lysine fractional abundance of quantified proteasome subunits show slowed turnover with marizomib. All data are mean ± SEM with $n = 5$ males. *p value < 0.05; **p value < 0.01; ***p value < 0.001.

 © The Author(s)
